# Extracellular vesicles from anoxia preconditioned mesenchymal stem cells alleviate myocardial ischemia/reperfusion injury

**DOI:** 10.18632/aging.202611

**Published:** 2021-02-12

**Authors:** Chengyu Mao, Dongjiu Li, En Zhou, Erhe Gao, Tiantian Zhang, Shufang Sun, Lin Gao, Yuqi Fan, Changqian Wang

**Affiliations:** 1 Shanghai Ninth People’s Hospital affiliated to Shanghai Jiao Tong University School of Medicine, Shanghai, China; 2 Lewis Katz School of Medicine, Temple University, Philadelphia, PA 19122, USA

**Keywords:** myocardial ischemia reperfusion injury, mesenchymal stem cells, extracellular vesicles, pyroptosis-induced apoptosis, GSDMD deficiency

## Abstract

Extracellular vesicles (EVs) produced by anoxia-preconditioned mesenchymal stem cells (MSCs) may afford greater cardioprotection against myocardial ischemia-reperfusion injury (MIRI) than EVs derived from normoxic MSCs. Here, we isolated EVs from mouse adipose-derived MSCs (ADSCs) subjected to anoxia preconditioning or normoxia and evaluated their ability to promote survival of mouse cardiomyocytes following MIRI *in vivo* and anoxia/reoxygenation (AR) *in vitro*. Injection of anoxia-preconditioned ADSC EVs (Int-EVs) reduced both infarct size and cardiomyocyte apoptosis to a greater extent than normoxic ADSC EVs (NC-EVs) in mice subjected to MIRI. Sequencing EV-associated miRNAs revealed differential upregulation of ten miRNAs predicted to bind thioredoxin-interacting protein (TXNIP), an inflammasome- and pyroptosis-related protein. We confirmed direct binding of miRNA224-5p, the most upregulated miRNA in Int-EVs, to TXNIP and asserted through western blotting and apoptosis assays a critical protective role for this miRNA against AR-induced cardiomyocyte death. Our results suggest that ischemia-reperfusion triggers TXNIP-induced inflammasome activation in cardiomyocytes, which leads to apoptosis rather than pyroptosis due to low basal levels of the pyroptosis executioner protein gasdermin D in these cells. The antiapoptotic effect of EV-associated miRNA224-5p would in turn result from TXNIP downregulation, which prevents caspase-1-mediated degradation of GATA4 and sustains the expression of Bcl-2.

## INTRODUCTION

Myocardial ischemia-reperfusion injury (MIRI) occurs upon restoration of blood flow (reperfusion therapy) following ischemia [1]. Despite great advancements in pharmacological and interventional revascularization therapies, MIRI remains a major challenge in clinical practice [2, 3]. The molecular mechanism behind MIRI involves secondary oxidative stress damage triggered by excessive production of mitochondrial reactive oxygen species (mROS) upon revascularization of the infarcted artery [4, 5]. Sustained and excessive mROS production is a common trigger of pyroptosis, which occurs after sequential activation of caspase-1 and gasdermin D (GSDMD) at the inflammasome. This results from mROS-mediated upregulation of thioredoxin-interacting protein (TXNIP), which represses the activity of hypoxia-inducible factor-1, hence weakening myocardial tolerance to anoxia and inducing inflammasome formation [6]. The ensuing inflammatory response and eventual cell demise both contribute to the progression of MIRI [7]. Once activated, caspase-1 also cleaves and degrades GATA binding protein 4 (GATA4) [8], a zinc finger transcription factor fundamentally involved in cardiomyocyte survival (apoptosis suppression) via transcriptional regulation of Bcl-2 expression. In this way, GATA4 functions as a link between pyroptosis and apoptosis to regulate programmed cell death in the myocardium. Accordingly, and despite many details remaining unclear, therapeutics targeting pyroptosis were shown to protect the heart from sterile inflammation, apoptosis, and dysfunction triggered by ischemia/reperfusion (I/R) injury [9, 10].

Extracellular vesicles (EVs) are cell-derived, membrane-bound spherical structures of 30-2,000 nm in diameter, released by almost all types of cells, including mesenchymal stem cells (MSCs). Main EV types include exosomes and microvesicles, which can modulate nonrestricted or cell-specific intercellular communication by transferring proteins, mRNAs, and miRNAs [11, 12]. EVs’ contents usually reflect the molecular makeup of the source cell and can modulate in target cells various physiological and pathological processes, including cell proliferation, differentiation, secretion, migration, and death. In turn, changes in the cell microenvironment and physiological state can enhance or weaken the biological function of secreted EVs.

EVs have been reported to be important regulators of various cardiovascular diseases [13]. For example, EVs derived from MSCs have the potential of protecting cardiomyocytes from MIRI and non-specific inflammation [14]. Cardiac fibroblast-derived EVs facilitate pathological cardiac hypertrophy via activating the renin angiotensin system in cardiomyocytes [15], whereas EVs derived from cardiomyocytes contribute to cardiac fibrogenesis via myocyte-fibroblast cross-talk [16]. This implies that the cell source of EVs greatly determines their modality of action and functional outcome.

Since anoxia preconditioning can enhance cardiomyocyte tolerance to an oxygen-deficient environment, inflammation, and oxidative stress [17, 18], we hypothesized that EVs derived from anoxia-preconditioned MSCs will be more effective against MIRI-associated cardiomyocyte death than EVs derived from normoxic MSCs. To test this hypothesis, we isolated EVs derived from mouse adipose-derived mesenchymal stem cells (ADSCs) subjected to either normoxia or anoxia preconditioning and tested their ability to counteract cardiomyocyte death following MIRI *in vivo* and anoxia/reoxygenation (AR) *in vitro*. Specifically, we analyzed morphological and molecular changes associated with I/R-induced pyroptosis and apoptosis in cardiomyocytes and unmasked a potential role for anoxia-induced, EV-associated miRNAs in cardiomyocyte survival.

## RESULTS

### Characterization of ADSCs and ADSC-derived EVs

**Figure 1:**
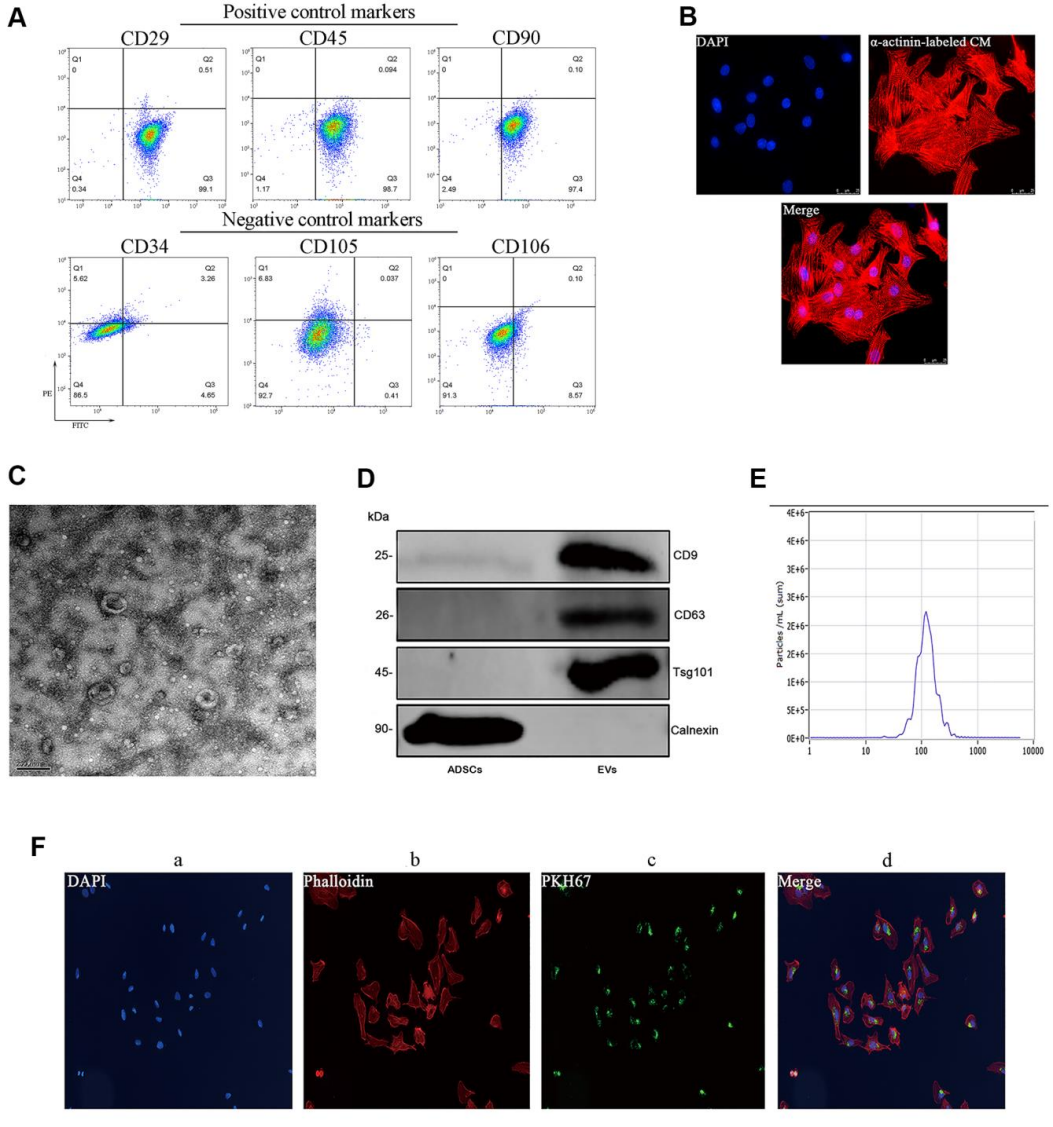
**Characterization of mouse ADSCs and ADSC-derived EVs.** (**A**) Analysis of cell surface markers (CD29, CD45, and CD90) in isolated mouse ADSCs; CD34, CD105, and CD106 were used as negative controls. (**B**) The purity of neonatal mouse cardiomyocytes was estimated to be higher than 95% based on α-actinin staining results. (**C**) TEM characterization of EVs. (**D**) Western blot examination of EV markers. Calnexin was used as negative control. (**E**) NanoSight’s light scattering intensity-based size distribution of isolated EVs. (**F**) EV tracer (PKH67) assay demonstrating successful EV internalization by cardiomyocytes.

Successful isolation of ADSCs from mouse adipose tissue was verified through flow cytometry, which demonstrated positive expression (>95%) of the cell surface markers CD29, CD45, and CD90 and low/negative expression of CD34, CD105, and CD106 (Figure 1A). Subsequently, ADSC-derived EVs (ADSC-EVs) were obtained through sequential centrifugation and characterized using both a Nanosight device and transmission electron microscopy (TEM). These analyses indicated that the collected vesicles were mainly exosomes, with a median average size of 115 nm (range: 50-150 nm) (Figure 1C–1E). The isolated vesicles expressed three EV-associated markers, i.e. CD9, CD63, and tumor susceptibility gene 101 (TSG101) and were negative for calnexin (Figure 1D). Internalization of PKH67-labeled EVs by cultured neonatal mouse cardiomyocytes was confirmed by fluorescence microscopy (Figure 1F).

### ADSC-EVs reduce pyroptosis and apoptosis in cardiomyocytes subjected to anoxia/reoxygenation

**Figure 2:**
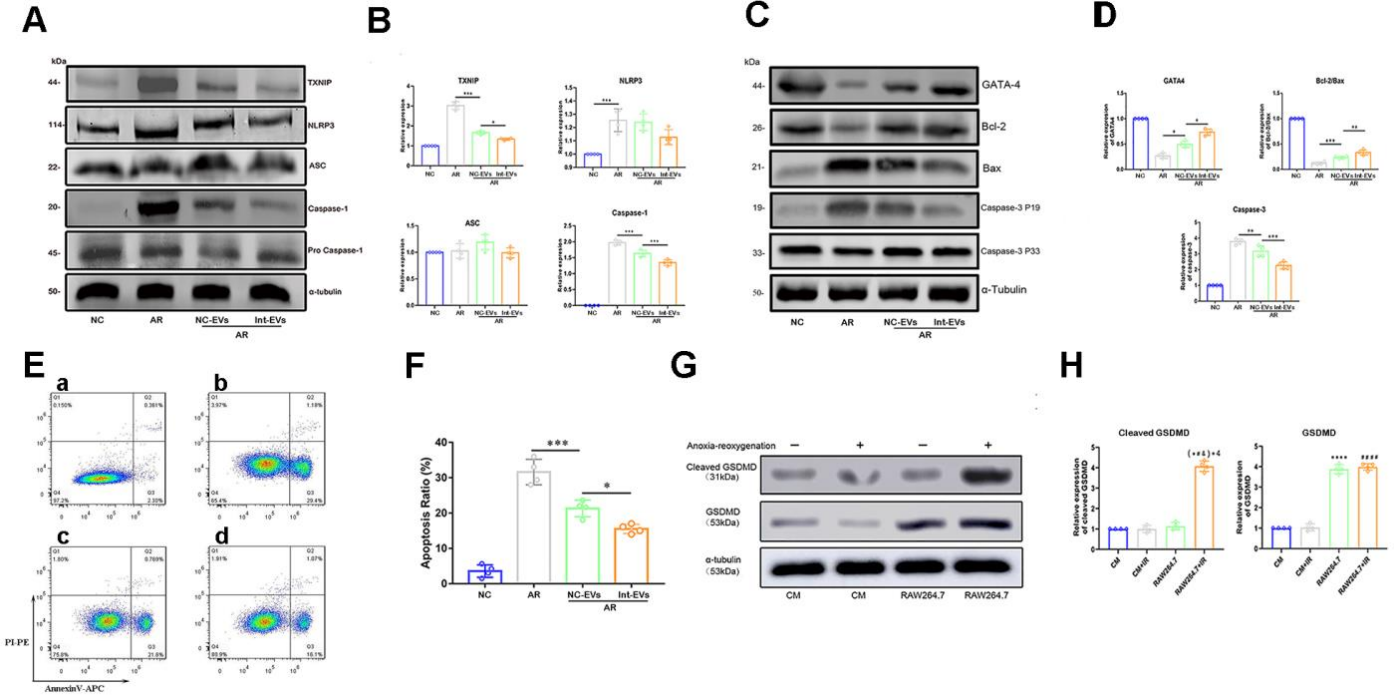
**Analysis of pyroptosis and apoptosis markers in EV-treated, AR-exposed cardiomyocytes.** (**A**, **B**) Western blot analysis of TXNIP and cleaved-caspase-1 in AR-exposed cardiomyocytes incubated with EVs derived from normoxic (NC-EVs) or anoxic (Int-EVs) ADSCs. The NC-EVs group was compared with the control AR group and the Int-EVs group was compared with the NC-EVs group (*P < 0.05, **P < 0.01, *** P < 0.001, **** P < 0.0001, n = 4). (**C**, **D**) Western blot analysis of BAX, cleaved-caspase-3, GATA4, and Bcl-2 in cardiomyocytes treated with AR. Comparisons were made between NC-EVs and AR and between Int-EVs and NC-EVs groups (*P < 0.05, **P < 0.01, *** P < 0.001, **** P < 0.0001, n = 4). (**E**, **F**) Apoptosis detection in AR-exposed cardiomyocytes (Annexin V/PI assay). The NC-EVs group was compared with the AR group and the Int-EVs group was compared with the NC-EVs group (*P < 0.05, **P < 0.01, *** P < 0.001, **** P < 0.0001, n = 4). (**G**, **H**) Relative expression of cleaved GSDMD and total GSDMD in cardiomyocytes and RAW264.7 cells treated with or without AR (*P < 0.05, **P < 0.01, *** P < 0.001, **** P < 0.0001, compared with the control CM group; n = 4); (#P < 0.05, ##P < 0.01,###P < 0.001, #### P < 0.0001, compared with the CM + AR group; n = 4); (&P < 0.05, &&P < 0.01, &&& P < 0.001, &&&&P < 0.0001, compared with the RAW264.7 group; n = 4).

To examine whether ADSC-EVs can prevent or attenuate MIRI-induced pyroptosis in cardiomyocytes, pyroptosis markers were detected by western blotting in cultured neonatal mouse cardiomyocytes which were subjected to anoxia/reoxygenation (AR) and were exposed to EVs derived from normoxic ADSCs (NC-EVs) or to EVs derived from anoxia-preconditioned ADSCs (Int-EVs) 2h prior to AR. As shown in Figure 2A, 2B, exposure to NC-EVs decreased the expression of both TXNIP and cleaved-caspase-1 (TXNIP, p=0.0007; cleaved-caspase1, p=0.0002; compared to control AR). However, the downregulation of the two pyroptosis markers was significantly higher after exposure to Int-EVs (TXNIP, p=0.0194; cleaved-caspase-1, p=0.0007; compared to NC-EVs).

Active caspase-1 can cleave and deactivate GATA4, a key transcription factor regulating cardiac development and cardiomyocyte survival [8]. To evaluate whether ADSC-EVs can prevent both GATA4 downregulation and apoptosis in AR-treated cardiomyocytes, we conducted western blotting and Annexin V/PI assays following the manufacturer's instructions. As shown in Figure 2C, 2D, exposure to NC-EVs decreased the expression of cleaved-caspase-3 (a downstream target of cleaved caspase-1) and upregulated both GATA4 and the Bcl-2/Bax ratio (GATA4, p=0.036; Bcl-2/Bax, p=0.0007; cleaved-caspase-3, p=0.0058; compared to AR). Once again, however, the observed changes were significantly larger in Int-EV-treated cells (GATA4, p=0.0106; Bcl-2/Bax, p=0.0022; cleaved-caspase-3, p=0.0003; compared to NC-EVs). Meanwhile, as shown in Figure 2E, 2F, Annexin V-PI assays demonstrated a reduced rate of apoptosis after cardiomyocyte exposure to either Int-EVs or NC-EVs, with Int-EVs eliciting in turn a larger anti-apoptotic effect (NC-EVs vs AR, p=0.0002; Int-EVs vs NC-EVs, p=0.0143).

### GSDMD expression in mouse cardiomyocytes is low and unaffected by AR

Caspase-1 mediated cleavage of GSDMD, a pore-forming protein, is a key event in the onset of pyroptosis triggered by the TXNIP/NLRP3 inflammasome pathway. Since AR-mediated TXNIP activation in cardiomyocytes fails to activate pyroptosis, we investigated the effect of AR on GSDMD expression in both mouse neonatal cardiomyocytes and myeloid RAW264.7 cells, which are known to express high GSDMD levels. As shown in Figure 2G, 2H, no differences in cleaved GSDMD expression were found between control (CM) and AR-exposed (CM + AR) cardiomyocytes (p=0.991). Meanwhile, stimulated with AR, expression of cleaved GSDMD in RAW264.7+AR group was significantly higher than other comparisons (p<0.0001 for all comparisons). In addition, total GSDMD expression was found to be much higher in RAW264.7 cells than in cardiomyocytes (p<0.0001).

### ADSC-EVs ameliorate myocardial damage in a mouse model of MIRI

**Figure 3:**
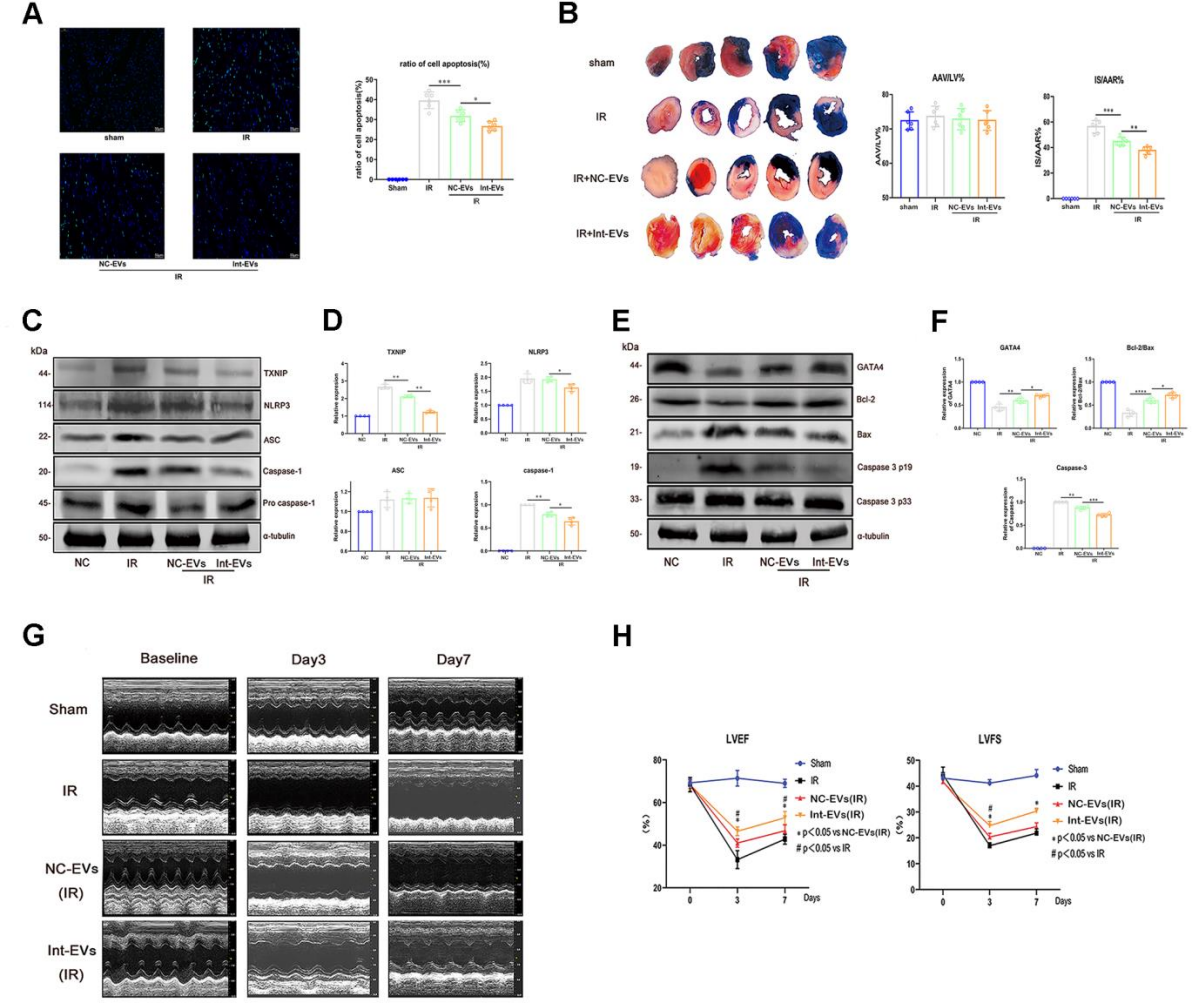
**Cardioprotective effects of ADSC-derived EVs in a mouse model of MIRI.** (**A**) Apoptosis (TUNEL) assay in mouse myocardial sections obtained 12h after reperfusion. The NC-EVs group was compared with the IR group and the Int-EVs group was compared with the NC-EVs group (Image below: 400× magnification; *P < 0.05, **P < 0.01, *** P < 0.001, **** P < 0.0001, n = 6). (**B**) Assessment of infarct size (IS) and area at risk (AAR) in mice subjected to MIRI. Results were compared between the NC-EVs and the IR groups and between the Int-EVs and the NC-EVs groups (10× magnification; IR: ischemia reperfusion; TTC: triphenyltetrazolium chloride; AAR: *P < 0.05, **P < 0.01, *** P < 0.001, **** P < 0.0001, n = 6). (**C**, **D**) Western blot analysis of TXNIP, cleaved-caspase-1, and NLRP3 in infarcted mouse myocardial tissue. Data were compared between the NC-EVs and the IR groups and between the Int-EVs and the NC-EVs groups (*P < 0.05, **P < 0.01, *** P < 0.001, **** P < 0.0001, n = 4). (**E**, **F**) Western blot analysis of BAX, cleaved-caspase-3, GATA4, and Bcl-2 expression in infarcted mouse myocardial tissue. Data were compared between the NC-EVs and the IR groups and between the Int-EVs and the NC-EVs groups (*P < 0.05, **P < 0.01, *** P < 0.001, **** P < 0.0001, n = 4). (**G**, **H**) Cardiac function assessment. Echocardiography was used to examine EF and FS immediately after sham or MIRI surgery (baseline) and on the 3^rd^ and 7^th^ day after reperfusion (#P < 0.05, ##P < 0.01, ### P < 0.001, #### P < 0.0001, NC-EVs vs IR; n = 7). (*P < 0.05, **P < 0.01, *** P < 0.001, **** P < 0.0001, Int-EVs vs NC-EVs; n = 7).

To evaluate whether ADSC-EVs can protect against MIRI-induced myocardial damage *in vivo*, cardiac infarct size (IS) and area at risk (AAR) were assessed in mice intravenously injected with ADSC-EVs before MIRI surgery. As shown in Figure 3B, AAR/LV values were similar between the sham, IR (MIRI), and IR + NC-EVs groups (re-ligating left coronary artery prior to Evens blue stain to calculate AAR/LV values). In contrast, the NC-EVs and Int-EVs groups exhibited significantly reduced post-MIRI IS compared to the IR group, with the Int-EVs group showing again the most significant effect (NC- EVs vs IR; p=0.0007; Int- EVs vs NC-EVs; p=0.0028).

### *In vivo* infusion of ADSC-EVs inhibits MIRI-induced cardiomyocyte death

To confirm that ADSC-EVs attenuate MIRI by promoting cardiomyocyte survival, apoptosis and pyroptosis were examined in myocardial tissue *ex-vivo* following intravenous infusion of ADSC-EVs and MIRI surgery in mice. TUNEL assay results showed that administration of NC-EVs reduced the rate of apoptosis in the myocardium (NC-EVs vs IR; p=0.0003), an effect that was, in turn, significantly enhanced after injection of Int-EVs (Int-EVs vs NC-EVs, p=0.0141) (Figure 3A). As shown in Figure 3E and 3F, these pro-survival effects were paralleled by decreased expression of cleaved-caspase-3, upregulated expression of GATA4, and increased Bcl-2/Bax ratio in myocardial cells (NC-EVs vs IR: GATA4, p=0.0026; Bcl-2/Bax, p=0.0001; cleaved-caspase-3, p=0.0011; Int-EVs vs NC-EVs: GATA4, p=0.0242; Bcl-2/Bax, p=0.027; cleaved-caspase-3, p=0.0006).

To evaluate whether ADSC-EVs could also inhibit MIRI-related pyroptosis of cardiac cells, pyroptosis pathway markers were detected in the excised hearts. As shown in Figure 3C, 3D, the expression of both TXNIP and cleaved-caspase-1 decreased upon pre-treatment with NC-EVs (TXNIP, p=0.009; cleaved-caspase1, p=0.0078; compared to IR). Of note, further reductions in TXNIP, cleaved-caspase-1, and NLRP3 levels were detected in samples from Int-EV-treated mice (TXNIP, p=0.0042; NLRP3, p=0.0341; cleaved-caspase1, p=0.0287; compared to NC-EVs).

### ADSC-EVs improve cardiac function after MIRI

Echocardiography on days 3 and 7 post-MIRI revealed that ejection fraction (EF) and left ventricular fractional shortening (LVFS) were significantly improved in mice that received ADSC-EVs (Figure 3G, 3H), and especially Int-EVs (EF day 3: NC-EVs vs IR, p=0.020; Int-EVs vs NC-EVs, p=0.036; FS day 3: NC-EVs vs IR, p=0.033; Int-EVs vs NC-EVs, p=0.015; EF day 7: NC-EVs vs IR, p=0.022; Int-EVs vs NC-EVs, p=0.017; FS day 7: NC-EVs vs IR, p=0.058; Int-EVs vs NC-EVs, p=0.014).

### Anoxic preconditioning upregulates miR-224-5p, a potential TXNIP regulator, in ADSC-EVs

**Figure 4:**
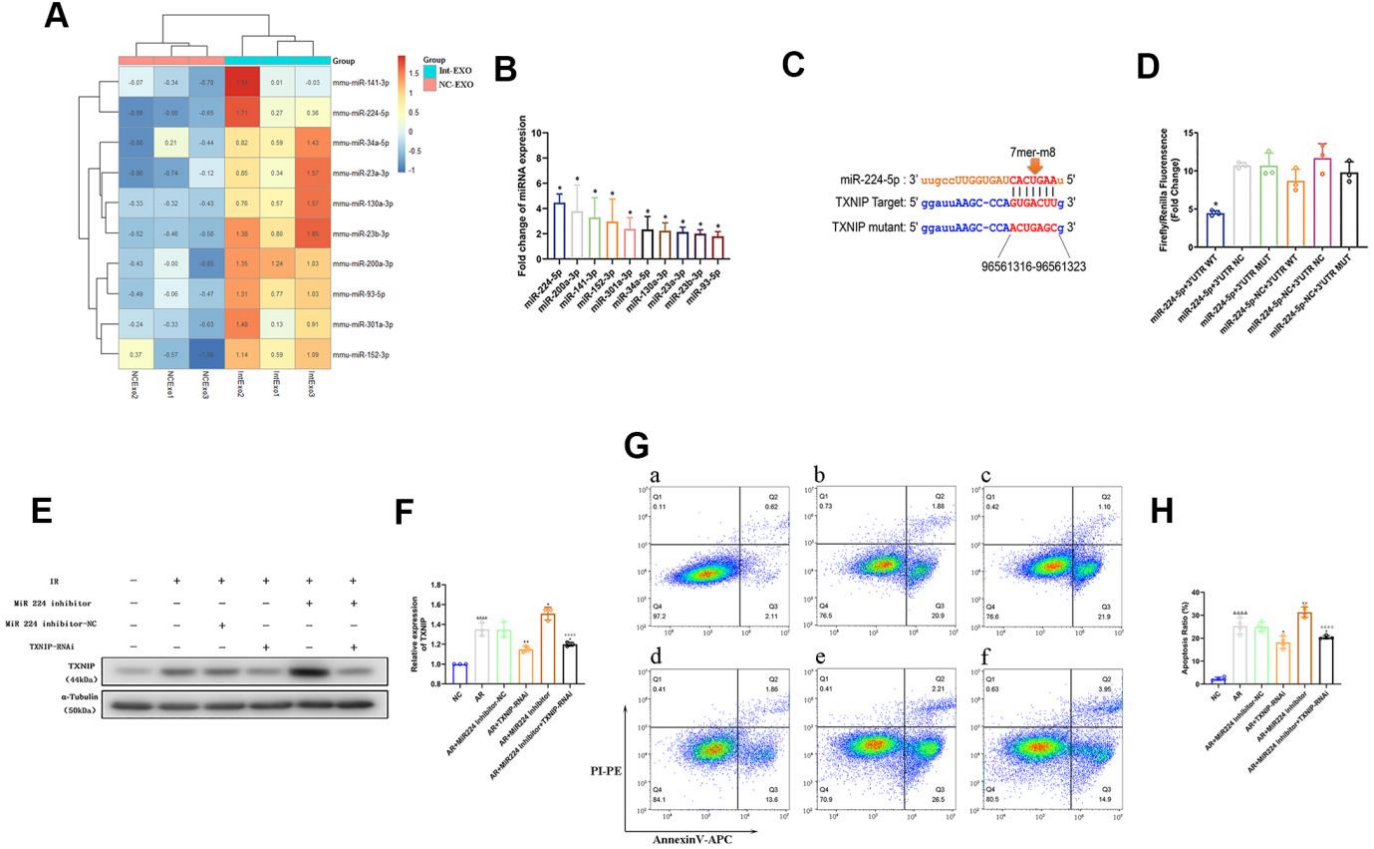
**Anoxia-induced, EV-associated miR-224-5p inhibits I/R-mediated apoptosis in cardiomyocytes by downregulating TXNIP.** (**A**) MicroRNA sequencing analysis in ADSC-EVs. A total of 41 miRNAs were upregulated in Int-EVs compared to NC-EVs. Ten of them (shown in the heatmap) were predicted to associate with TXNIP. (**B**) Validation of differential EV-associated miRNA expression through qRT-PCR. U6 snRNA served as internal reference (*P < 0.05, compared with NC-EVs; n=3). (**C**) Dual-luciferase reporter assay. HEK 293 T cells were co-transfected with miR-224-5p mimics and PGL3 luciferase reporter plasmids containing wild-type or mutated TXNIP 3′UTR. An NC-TXNIP 3′UTR served as control. (**D**) Dual-luciferase reporter assay analysis (*P < 0.05, miR-224-5p + TXNIP WT 3′UTR vs mutated- and NC-3’UTR groups; n=3). (**E**) Analysis of TXNIP and miR224-5p expression in AR-treated mouse cardiomyocytes transfected with a miR224-5p inhibitor and/or TXNIP-targeting siRNAs (TXNIP-RNAi). (**F**) Statistical analysis of data from experiments like those shown in (**E**); (&P < 0.05, &&P < 0.01, &&& P < 0.001, &&&&P < 0.0001, compared with the control group; n = 3; *P < 0.05, **P < 0.01, *** P < 0.001, **** P < 0.0001, compared with the AR group; n = 3; #P < 0.05, ##P < 0.01, ### P < 0.001, #### P < 0.0001, compared with the AR + inhibitor group; n = 3). (**G**) Representative images of Annexin V/PI-stained cardiomyocytes transfected with a miR224-5p inhibitor and/or TXNIP-targeting siRNAs prior to AR. a) NC: non-AR control; b) AR; c) Inhibitor-NC + AR: miR224-5p inhibitor (negative control) pre-treatment followed by AR; d) TXNIP-RNAi + AR: TXNIP-RNAi pre-treatment followed by AR; e) Inhibitor + AR: miR224-5p inhibitor pre-treatment followed by AR; f) Inhibitor + TXNIP-RNAi + AR: miR224-5p inhibitor plus TXNIP-RNAi pre-treatment followed by AR. (**H**) Statistical analysis of Annexin V/PI staining experiments. (&P < 0.05, &&P < 0.01, &&& P < 0.001, &&&&P < 0.0001, compared to control; n = 4; *P < 0.05, **P < 0.01, *** P < 0.001, **** P < 0.0001, compared to AR; n = 4; #P < 0.05, ##P < 0.01, ### P < 0.001, #### P < 0.0001, compared to miR224-5p inhibitor + AR; n = 4).

To explore the mechanism underlying the higher inhibitory efficacy of Int-EVs on AR-mediated cardiomyocyte cell death, microRNA (miRNA) sequencing was performed on NC-EVs and Int-EVs. A total of 41 miRNAs were upregulated in Int-EVs compared to NC-EVs (Supplementary Table 1). Among those, 10 were predicted to associate with TXNIP by the TargetScan and miRanda algorithms in The Encyclopedia of RNA Interactomes (ENCORI) database. Differential expression of these miRNAs was confirmed by real-time PCR (Figure 4A, 4B). Among the 10 upregulated miRNAs predicted to associate with TXNIP, miR-224-5p was selected for further study because its upregulation was the most significant. Dual-luciferase reporter assay results showed that the fluorescence ratio was significantly reduced from HEK 293T cells upon co-transfection with miR-224-5p mimics and TXNIP WT-3’ UTR, compared to the NC and mutant 3’ UTR controls (p=0.0011) (Figure 4C, 4D). These results indicated that miR-224-5p inhibits the translation of TXNIP by targeting the 3’ UTR region of its mRNA.

### miR-224-5p ameliorates AR-induced cardiomyocyte apoptosis by targeting TXNIP

To assess whether miR-224-5p can attenuate AR-induced apoptosis in cardiomyocytes by inhibiting TXNIP, western blotting and Annexin/V-PI assays were performed on AR-exposed neonatal mouse cardiomyocytes pre-treated with a miR-224-5p inhibitor or a TXNIP-targeted siRNA (TXNIP-RNAi) to silence TXNIP expression. As shown in Figure 4E, 4F, transfection with the miR-224-5p inhibitor decreased miR-224-5p and increased TXNIP expression relative to untransfected control (AR) cells (p=0.022). AnnexinV/PI staining results (Figure 4G, 4H) indicated, as expected, significant induction of apoptosis in AR-treated cardiomyocytes, compared to non-AR control (CON) cells (p<0.0001). Of note, this effect was significantly enhanced by inhibition of miR-224-5p (p=0.0115, compared to AR). In contrast, inhibition of TXNIP in cardiomyocytes undergoing AR significantly reduced the apoptosis rate (p=0.0025, compared to AR). Importantly, the pro-apoptotic effect elicited by miR-224-5p suppression could be reversed by co-transfection with TXNIP-RNAi (p <0.0001). These results demonstrated that miR-224-5p inhibits apoptosis of cardiomyocytes undergoing AR by targeting TXNIP.

## DISCUSSION

Based on excellent biocompatibility, low immunogenicity, and ability to cross the blood-brain barrier, EVs are considered as efficient nanoscale vehicles for tissue- or cell-targeted delivery of small molecules and nucleic acids. Mounting evidence suggests also that EVs possess great potential to deliver cardioprotective non-coding RNAs, cytokines, and small-molecule drugs for the treatment of ischemic heart disease [19, 20]. Although solid advances have been made, clinical implementation of EV-based therapies still faces some concerns: (1) EV isolation methods have not yet been standardized to meet clinical needs; (2) biosafety concerns have arisen regarding the use of synthetic vesicles and technologies to edit EVs’ contents; (3) clinical trials have not yet fully assessed the potential side-effects derived from long-term use of EVs [21, 22]. Thus, our aim was to improve the therapeutic efficacy of MSC-derived EVs against I/R-induced cardiomyocyte death by using a safe anoxia-preconditioning method *in vitro*, and to explore the bases of EVs’ protective effects.

Remote ischemic preconditioning (RIPC) is defined as the transient, repeated stimulation of remote tissues or organs with brief episodes of ischemia that serves to protect a target organ against prolonged I/R injury [23, 24]. Highlighting a potential mechanism for RIPC efficacy, a higher therapeutic potential has been demonstrated for EVs produced by MSCs cultured under hypoxic, rather than normoxic, conditions [25]. For instance, EVs released from hypoxia-preconditioned MSCs were recently proposed as paracrine effectors of traumatic spinal cord injury repair [26] and elicited cardioprotection in a rat model of MIRI [27]. Thus, our study used a mouse model of MIRI to investigate whether an *in vitro* RIPC protocol could enhance the cardioprotective potential of ADSC-derived EVs. Our results demonstrated that EVs produced by anoxia-preconditioned mouse ADSCs (Int-EVs) mediate greater suppression of I/R-induced cardiomyocyte death than EVs derived from normoxic ADSCs (NC-EVs). Importantly, we identified 41 miRNAs differentially enriched in EVs generated by anoxia-preconditioned ADSCs, ten of which are putative regulators of inflammasome activation based on predicted binding affinity for TXNIP. We then focused on the most upregulated miRNA, i.e. miR-224-5p, and verified both direct binding to TXNIP and a critical role for this interaction in the inhibition of AR-induced cardiomyocyte death.

The zinc finger transcription factor GATA4 has an essential cardioprotective role and is a key mediator of the adaptability of the myocardium to perfusion pressure [28]. Indeed, research has shown that forced upregulation of GATA4 attenuates experimentally-induced apoptosis in cardiomyocytes [29], primarily through transcriptional induction of Bcl-2 expression [30–32]. Consequently, GATA4 has emerged as a promising therapeutic target for ischemic cardiomyopathy [33]. Here, we showed that exposure to ADSC-EVs, and especially to EVs derived from anoxia-preconditioned ADSCs, attenuates I/R-induced apoptosis of cardiomyocytes both *in vitro* and *in vivo*, in parallel with upregulation of both GATA4 and the Bcl-2/Bax ratio.

Activation of caspase-1 links pyroptosis with inflammation by mediating proteolytic activation of IL-1β, IL-18, and GSDMD [34]. In addition, active caspase-1 can cleave and inactivate GATA4 by binding to two functional sites, YMAD^168^ within the major transcription activation domain and WRRD^230^ within the first zinc finger [8]. Based on this knowledge, our results suggest that by delivering TXNIP-targeting miRNAs, Int-EVs inhibit I/R-induced, inflammasome-dependent pyroptosis and prevent also subsequent apoptosis by restricting caspase-1-mediated cleavage and inactivation of GATA4 in cardiomyocytes.

Until a few years ago, the various types of programmed cell death were considered to be discrete and unrelated, with apoptosis and pyroptosis being described as independent phenomena with characteristic morphological, mechanistic, and functional features [35]. Although distinct molecular events (cell shrinkage and formation of vesicular/apoptotic bodies in apoptosis; inflammasome activation, membranolysis and release of inflammatory cytokines in pyroptosis) distinguish indeed the two cell death programs [36, 37], research has shown that cellular levels of GSDMD, the executioner of pyroptosis, can determine whether caspase-1 activation leads to pyroptosis or triggers apoptosis instead. Active caspase-1 cleaves GSDMD between its N-terminal and C-terminal domains. Upon translocation to the plasma membrane, activated GSDMD forms pores to induce cell death [38]. Caspase-1 has been long considered mainly an inflammation-related protein, probably because most studies conducted on inflammasomes used, as research models, inflammatory (e.g. myeloid) cells which express abundant GSDMD [39]. However, earlier studies indicated that upon deficiency of GSDMD active caspase-1 could trigger cell apoptosis through BH3-interacting domain death agonist (Bid) and other pathways [40, 41]. Therefore, caspase-1 is at the crossroads of pyroptosis and apoptosis since its activation can induce apoptosis instead of pyroptosis in cell types that do not or only weakly express GSDMD [42].

**Figure 5:**
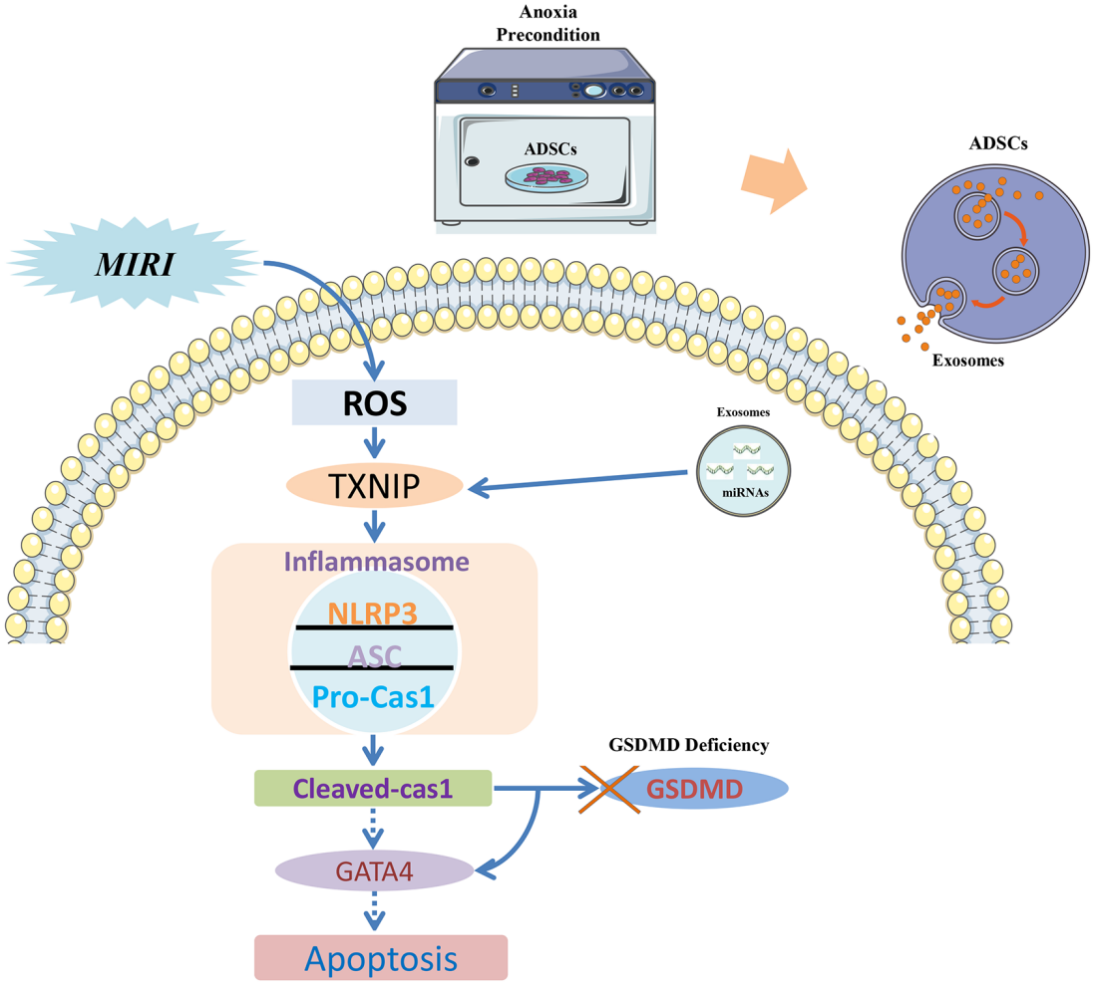
**Signaling pathway diagram.** Myocardial ischemia-reperfusion injury (MIRI) increases ROS production, which promotes the association of TXNIP with NLRP3 to activate the inflammasome. Because cardiomyocytes show low levels of the pyroptosis effector protein GSDMD, the apoptotic pathway becomes instead activated due to caspase-1-mediated degradation of the transcription factor GATA4 and consequent downregulation of the anti-apoptotic gene Bcl-2. EVs derived from ADSCs exposed to anoxic preconditioning exert significant cardioprotective effects against MIRI due to a distinct abundance of miRNAs targeting TXNIP. TXNIP downregulation impedes caspase-1 activation and GATA4 degradation, which therefore sustains Bcl-2 expression and prevents MIRI-induced apoptosis of cardiomyocytes.

A signaling pathway diagram that describes caspase-1-induced apoptosis and its prevention by Int-EVs is presented in the Figure 5. Consistent with previous results [40], our study revealed that mouse cardiomyocytes show a relatively low expression of total GSDMD protein and that cleavage of GSDMD was also inapparent upon activation of the caspase-1/pyroptosis pathway. This would suggest that upon GSDMD deficiency, the last stage of pyroptosis, namely membranolysis, is blocked. Thus, the main manifestation of MIRI in cardiomyocytes is apoptosis, even though the observed cleavage of GATA4 indicates that the pyroptosis pathway was activated. This mechanism is supported by the present evidence that upon TXNIP inhibition by Int-EV-associated miRNAs, reduced activation of the inflammasome and decreased cleavage of caspase-1 are paralleled by downregulation of both GATA4 and its direct target Bcl-2.

In conclusion, our study indicates that EVs produced by ADSCs subjected to anoxic preconditioning exert a more significant cardioprotective effect against MIRI than EVs derived from normoxic ADSCs. This could be attributed to the presence of a higher load of miRNAs targeting TXNIP in EVs generated by anoxia-exposed ADSCs. Our molecular analyses also suggest that TXNIP-mediated activation of the pyroptosis pathway in cardiomyocytes exposed to I/R determines apoptotic, rather than pyroptotic, cell death due to low GSDMD expression in these cells. Therefore, we propose that downregulation of TXNIP by EV-associated miRNAs prevents NLRP3 inflammasome activation and caspase-1-dependent cleavage of GATA4, which protects cells against apoptosis by sustaining the expression of Bcl-2. These findings provide new insights into the pathogenesis of MIRI and suggest that EVs derived from anoxia-preconditioned MSCs could help preserve myocardial integrity and function when applied at early MIRI stages.

## MATERIALS AND METHODS

### MIRI mouse model

All animal care and procedures were approved by the Shanghai Ninth People’s Hospital Institutional Ethics Committee (SH9H-2017-A39-1) and performed in accordance with the guidelines of the Directive 2010/63/EU of the European Parliament. Six-week-old male, specific-pathogen-free (SPF) wild-type (WT) C57BL/6 mice were purchased from the Experimental Animal Center of Shanghai Ninth People’s Hospital. Animals were fed with standard mouse chow and water ad libitum under SPF conditions (20-24° C; 50-60% humidity). All invasive procedures were performed under anesthesia, using first an anesthesia box with 3% isoflurane (Sinopharm Chemical Reagent Co. Ltd, Shanghai, China) for 1 min and administering thereafter 1.5-2% isoflurane through an anaesthetic mask. To minimize experimental variability, all animal procedures (MIRI, Evans blue/TTC stain) were performed by Prof. Erhe Gao. Mice were euthanized by carbon dioxide inhalation.

A total of 80 healthy WT C57BL/6 male mice (eight weeks old, ~24 g) were used for experiments. The MIRI model was generated as described in detail in Gao et al. [43] by ligating the left coronary artery (LCA) for 30 min with a slipknot, then releasing it to induce reperfusion. Successful MIRI was confirmed on the basis of dynamic electrocardiograph (ECG) changes (ST-segment elevation). Sham-operated mice, used as control, underwent the same procedure, with the exception that the knot on the LCA was left untied.

### Transthoracic echocardiography

Echocardiography was performed to assess ejection fraction (EF) and fractional shortening (FS) under 1.5-2% isoflurane anesthesia immediately after MIRI surgery (baseline) and on the 3^rd^ and 7^th^ days after reperfusion using a Vevo 770 high-resolution imaging system.

### Evaluation of area at risk and infarct size

After reperfusion for 4 h, the chest wall was reopened under 1.5-2% isoflurane anesthesia to expose the heart. The LCA was then re-ligated and the distal artery nipped, and 1% Evans Blue was injected through the ascending aorta until the non-infarction zone turned blue. The heart was harvested, washed in saline, and sliced horizontally into five pieces, from the left ventricular apex until the ligation site. All tissue pieces were incubated in 1% TTC for 20 min at 37° C. Image Pro Plus 6.0 was used to calculate infarct area and area at risk (AAR). AAR/left ventricle area (LV) ×100% and infarct size (IS) /AAR×100% were calculated.

### Isolation and culture of mouse adipose-derived stem cells

ADSCs were isolated from adipose tissue from C57BL/6 mice as previously described [44, 45]. In brief, adipose tissue isolated from the proximal limb was digested by 0.075% collagenase IV (Cat. 9001-12-1; Sigma, USA) for 60 min at 37° C in a mechanical horizontal rotator at a speed of 180 rpm. Collagenase IV was dissolved in complete medium containing DMEM/F12 (Cat. SH30023.01; Hyclone, USA) and 10 % fetal bovine serum (FBS; Cat. ST303302, PAN, Germany). After digestion, the samples were centrifugated at 200×g for 10 min and the cell pellets resuspended in complete medium (DMEM/F12 and 10% EV-free serum). The medium was then placed on 10 cm culture dishes in an incubator at 37° C in a 5% CO_2_ atmosphere. The extraction methods were based on previous references [44, 45] and remained undisturbed for three days prior to collection of EVs. Characterization of ADSCs was performed by flow cytometry analysis of CD29, CD45, and CD90 (positive cell surface markers) and CD34, CD105, and CD106 (negative controls).

### Isolation and culture of neonatal mouse cardiomyocytes

Cardiomyocytes were isolated from 1-2 day-old C57BL/6 mice as described previously [46]. In brief, mice were disinfected with a 75% ethanol solution and hearts quickly extracted and digested in 0.125% trypsin diluted with PBS at 4° C overnight. Following centrifugation at 200×*g* for 5 min, the cell pellet was resuspended and cultured in DMEM containing 10% FBS at 37° C in a 5% CO_2_ atmosphere for 1.5 h to allow for fibroblast attachment. Culture supernatants containing cardiomyocytes were then plated in 0.1% gelatin-coated dishes at a density of 1 × 10^6^ cells/mL. The purity of isolated cardiomyocytes was estimated by α-actinin staining.

### *In vitro* cardiomyocyte anoxia/reoxygenation model

An *in vitro* model of mouse cardiomyocyte anoxia/reoxygenation (AR) was established by incubating cells in oxygen-free, low-glucose DMEM in a controlled atmosphere (95% N2 and 5% CO_2_) for 2 h and reinstating afterwards normal oxygen levels and standard DMEM for 1 h.

### Anoxic preconditioning of ADSCs

ADSCs (1-2×10^6^ cells/100-mm cell-culture dish) were seeded in EV-free complete medium for 24 h. The medium was then exchanged for DMEM/F12 previously incubated with 100% N2 overnight to remove oxygen. Anoxic preconditioning was performed by exposing the cells to 5 cycles of anoxia (60 min in oxygen-free medium) with intermittent reoxygenation (30 min in normal DMEM/F12) in an anoxic chamber (Forma-1025 Anaerobic System, Thermo Fisher Scientific, USA). ADSCs were next incubated with serum-free medium in a normal incubation environment (21% O_2_, 5% CO_2_) and the supernatant was collected after 24 h.

### Isolation and characterization of EVs

EVs were isolated from cultured ADSCs (exposed or not to anoxic preconditioning) by differential centrifugation in conditioned media. In brief, the cell culture supernatant was centrifuged at 2,000×*g* for 30 min at 4° C to remove cell debris. The supernatant was then collected and centrifuged at 100,000×*g* for 70 min to precipitate EVs. The supernatant was discarded to remove contaminating proteins and EVs were resuspended in PBS and centrifuged at 100,000×*g* for 70 min. Aliquots of the EV precipitates were preserved at - 80° C to avoid repeated freeze/thaw cycles. EVs derived from non-preconditioned ADSCs are herein referred to as *NC-EVs*, whereas those derived from anoxia-preconditioned ADSCs are termed *Int-EVs*. EV size distribution and concentration were verified by a NanoSight NS300 instrument (Malvern Instruments, Malvern, UK) and EV morphology defined using transmission electron microscopy (TEM). Expression of the EV markers TSG101, CD9 (positive controls), and calnexin (negative control) was assessed by western blot [47].

### EV injections

Mice were randomly divided into 4 groups (n =20 mice per group): 1) sham (no MIRI; control); 2) MIRI; 3) NC-EVs (NC-EVs plus MIRI); and 4) Int-EVs (Int-EVs plus MIRI). On groups 3 and 4, EVs were administered at a dose of 10 μg per gram of body weight through tail vein injection prior to MIRI surgery.

### Analysis of EV uptake by cardiomyocytes

EVs and mouse cardiomyocytes were labeled with PKH67 (Cat. MINI67, Sigma-Aldrich, USA) and phalloidin (Cat. A12379s, ThermoFisher Scientific, USA), respectively, according to the manufacturers’ protocols. A total of 5 μL of PKH67-stained EVs was added to each cardiomyocyte culture, followed by a 2-h incubation to allow internalization by the cells. After washing twice with PBS, slides were fixed in 4% paraformaldehyde and cell nuclei were stained with DAPI. Fluorescent images were collected using an inverted microscope (BX63, Olympus, Japan) at 630x magnification.

### Cell transfection

293T cells were transfected with miR-224-5p mimics (Ribobio, Shanghai, China), Dual-Luciferase(DLU)-plasmid DNA using Lipofectamine 3000 transfection reagent (Cat. 11668019, Invitrogen, USA) following the manufacturer’s protocol. Mouse cardiomyocytes were transfected with a miR-224-5p inhibitor (RiboBio, Guangzhou, China) and small interfering RNAs targeting TXNIP (TXNIP-RNAi; RiboBio, Guangzhou, China) using RFect^PM^ transfection reagent (Cat.11011, BAIDAI, China) following the manufacturer’s protocol. Experiments were performed 24 h after transfection.

### Alpha-actinin immunofluorescence

Mouse cardiomyocytes cultured on confocal plates were fixed in 4% paraformaldehyde for 15 min, washed with PBS 3 times, and permeabilized with 0.5% Triton X-100 for 15 min. After blocking with primary antibody dilution buffer for 1 h at room temperature, a mouse anti-α-actinin primary antibody (1:1000; Cat. ab9465, Abcam, USA) was applied overnight at 4° C. After two washes in PBS, the cells were incubated with an Alexa Fluor 555 conjugate secondary antibody (1:1000; Cat. 4409, CST, USA) for 2 h at room temperature. Cell nuclei were stained with DAPI for 5 min at room temperature and fluorescence images collected on an inverted microscope (BX63, Olympus) at 630x magnification.

### Dual-luciferase reporter assay

HEK 293T cells were cultured in 24-well plates and co-transfected at ~70% confluence with miR 224-5p mimics and PGL3 luciferase plasmids containing WT, negative control (NC), or mutated TXNIP 3’UTR sequences. After 12 h, the cells were re-cultured in 96-well luciferase assay plates. The ratio of firefly to Renilla luciferase activity was detected 36 h later using a Dual-GLO^TM^ Luciferase Assay System (Cat. E2920, Promega, USA).

### Real-time PCR

Total EV-associated RNA was extracted with RNAiso Plus extraction reagent following the manufacturer's instructions (Cat. 9108, Takara, Dalian, China). Stem-loop primers (purchased from Ribobio Biotech) were used to produce cDNA from miRNA using a PrimeScript RT reagent Kit (Cat. RR047A, Takara). The miRNA cDNA was amplified using TB Green® Premix Ex Taq™ II (Cat. RR820L, Takara) on an ABI-7500 Real-Time PCR Detection System (Applied Biosystems, USA). U6 snRNA was used as internal control.

### Western blotting

Proteins were extracted from cardiac tissues and cardiomyocytes using Radio Immunoprecipitation Assay (RIPA). Ten mg of total protein was separated by 12% SDS-PAGE at 80 V for 1.5 h and then transferred to a PVDF membrane (Cat. IPVH00010, Millipore, USA) applying 300 mA for 1 h. After blocking, membranes were incubated with primary antibodies at 4° C overnight, washed, and treated with suitable secondary antibodies at room temperature for 1 h. Primary antibodies included rabbit anti-TSG101 (1:2000; Cat. ab125011, Abcam), rabbit anti-CD9 (1:2000; Cat. ab92726, Abcam), rabbit anti-TXNIP (1:1000; Cat. ab188865, Abcam), rabbit anti-ASC (1:1000; Cat. 67824, CST), rabbit anti-cleaved caspase-1 (1:1000; Cat. 89332, CST), rabbit anti-caspase-1 (1:1000; Cat. 24232, CST), rabbit anti-GATA4 (1:1000; Cat. ab134057, Abcam), rabbit anti-B-cell lymphoma-2 (Bcl-2; 1:1000; Cat. 3498, CST), rabbit anti-BCL2-associated X protein (BAX; 1:1000; Cat. 2772, CST), rabbit anti-caspase-3 (1:1000; Cat. 14220, CST), rabbit anti-α-actinin (1:1000; ab9465, Abcam), and rabbit anti-α-tubulin (1:1000; Cat. 2125, CST). Immunoreactive bands were visualized using an ECL substrate kit (Cat. ab65623, Abcam) and a two-color infrared fluorescence imaging system (Odyssey CLX, LICOR, USA). An anti-tubulin-α antibody was applied as internal control.

### Statistical analysis

Data analysis was performed using SPSS 19.0 software. The Shapiro-Wilk test was applied to assess whether data conformed to normal distribution. Pearson’s chi-square test (n ≥ 5) or Fisher’s exact test (n < 5) with subsequent multiple comparisons using chi-square testing with Bonferroni correction were used for categorical variables. One-way ANOVA with subsequent post-hoc multiple comparisons test (Student-Newman-Keuls test) was applied for continuous variables. The Kruskal–Wallis test was applied for nonparametric testing of multiple independent samples and a Dunn-Bonferroni test used for post hoc comparisons.

### Ethics approval

All animal procedures were approved by the Shanghai Ninth People’s Hospital institutional Ethics Committee (SH9H-2017-A39-1) and conducted in accordance with the guidelines of the Directive 2010/63/EU of the European Parliament.

### Availability of data and material

The data that support the findings of this study are available from the corresponding author upon reasonable request.

## Supplementary Material

Supplementary Table 1
